# Psychedelic interventions for major depressive disorder in the elderly: Exploring novel therapies, promise and potential

**DOI:** 10.1080/19585969.2025.2499458

**Published:** 2025-05-06

**Authors:** Ivona-Maria Tudorancea, Gabriela-Dumitrita Stanciu, Carla Torrent, Santiago Madero, Lucian Hritcu, Bogdan-Ionel Tamba

**Affiliations:** a Advanced Research and Development Center for Experimental Medicine “Prof. Ostin C. Mungiu” CEMEX, “Grigore T. Popa” University of Medicine and Pharmacy of Iasi, Iasi, Romania; b Bipolar and Depressive Disorders Unit, Hospital Clinic, Institute of Neuroscience, University of Barcelona, IDIBAPS, CIBERSAM, Barcelona, Spain; c Laboratory of Animal Physiology, Alexandru Ioan Cuza University of Iasi, Iasi, Romania; d Department of Pharmacology, Clinical Pharmacology and Algesiology, “Grigore T. Popa” University of Medicine and Pharmacy of Iasi, Iasi, Romania

**Keywords:** Psychedelic-assisted therapy, ageing and depression, mental health care, age-related comorbidities

## Abstract

The global population is ageing rapidly, with the number of individuals aged 60 and older reaching 1 billion in 2019 and expected to double by 2050. As people age, neuropsychological health often deteriorates, leading to a higher prevalence of age-related depression. Symptoms may include anxiety, apathy, mood instability, sadness, and, in severe cases, suicidal thoughts. Depression in the elderly is a widespread concern, and conventional treatments such as antidepressants are often limited by side effects, reduced efficacy, and complications arising from polypharmacy. In response, novel therapeutic approaches are being explored, including psychedelic interventions. Recent clinical and preclinical studies suggest that psychedelics could offer a promising treatment for major depressive disorder (MDD) in older adults. These compounds, known for their profound neurobiological effects, have gained attention for their potential to address depression where traditional therapies fall short. This review aims to examine the therapeutic promise of psychedelic substances, focusing on those that show potential for treating MDD in the elderly. We also explore the underlying mechanisms through which psychedelics may exert their effects and highlight the preclinical models that support their use. Finally, we address safety considerations and propose strategies to enhance the effectiveness and safety of psychedelics in future clinical trials, offering new hope for treating age-related depressive disorders.

## Introduction

Ageing populations have become a major public health concern, driven by increasing life expectancy and the rising prevalence of age-related comorbidities. Recent projections suggest that the global population aged 60 and older will double by 2050, growing from 1 billion to 2 billion. Furthermore, the number of individuals over 80 years old is expected to triple during the same period (World Health Organization 2024). This unprecedented demographic shift presents an important challenge for modern medicine, as ageing is frequently accompanied by a rising prevalence of chronic and debilitating conditions, among which depression stands out due to its poor prognosis. A specific form of depression observed in individuals aged 60 and older, termed late-life depression (LLD) or major depressive disorder (MDD), can manifest as either late-onset or an exacerbation of earlier depressive episodes. The prevalence of depression among older adults varies significantly worldwide, ranging from 8.2% to 63.0% (Manandhar et al. 2019; Hu et al. 2022). This wide variation is influenced by a combination of economic, cultural, and infrastructural factors unique to each region (Hall et al. 2022). In regions with higher economic development, such as Northern Europe and North America, access to healthcare services is more widespread, and public health policies are more comprehensive, enabling better management and treatment of mental health issues. Additionally, these areas tend to have lower stigma surrounding mental health, making it more likely for older adults to seek psychiatric help when necessary (Schnittker 2020; Yang et al. 2020). On the other hand, in less economically developed regions, such as parts of Africa and Asia, depression tends to be more prevalent (Hu et al. 2022). Limited access to healthcare services and poorer living conditions significantly contribute to the higher incidence of depression in these regions. Furthermore, the pressures related to basic survival—such as safety and access to food—often exacerbate the risk of depression among older adults (Cai et al. 2023; Jalali et al. 2024). Cultural factors and attitudes towards mental health, including the stigma associated with mental illnesses, also play a key role in shaping the prevalence of depression across different regions (Oldewage-Theron and Egal 2021). It is also important to note that much of the research on depression in older adults has primarily focused on individuals who are clinically diagnosed with depression, often overlooking those who experience depressive symptoms without a formal diagnosis. As Abdoli et al. (2022) point out, these individuals may still face significant psychological distress. Moreover, studies emphasise the difficulties in obtaining consistent global data due to variations in research methodologies and regional differences, which complicates the establishment of a clear and universally accepted prevalence rate for depression in older adults (Krishnamoorthy et al. 2020; Cai et al. 2023; Jalali et al. 2024). MDD in older adults is characterised by a diverse range of clinical symptoms, including insomnia, anhedonia, anxiety, lack of motivation, feelings of guilt, and a profound loss of interest in daily activities. This heterogeneous symptomatology highlights the need for personalised therapeutic approaches and comprehensive care strategies to address the unique mental health challenges faced by this growing demographic (Zhao et al. 2023). A timely and accurate diagnosis of depression in older adults is essential to prevent symptom progression and avoid severe complications, such as suicide and increased mortality rates. However, diagnosing depression in the elderly is often complicated by the overlap between depressive symptoms and other age-related conditions. One of the major diagnostic challenges is distinguishing between depression and the psychological and physical manifestations of frailty, a condition commonly observed in ageing individuals. Frailty is characterised by unintentional weight loss, fatigue, reduced walking speed, low activity levels, and generalised weakness—all of which can mimic depressive symptoms. These overlapping features underscore the importance of comprehensive clinical assessments that consider both psychological and physiological factors in order to facilitate early intervention and effective management of depression in older adults. Recent studies suggest that MDD and frailty are not simply two separate comorbidities that can be managed independently. Rather, they are often interconnected manifestations of an underlying disorder, with each condition exacerbating the other. Frailty can act as a trigger for depressive symptoms, while depression can accelerate the progression of frailty, creating a vicious cycle of declining physical and mental health. This bidirectional relationship highlights the importance of integrated therapeutic approaches that address both the physical and mental health needs of older adults simultaneously, which is crucial for breaking this cycle and improving overall health outcomes (Brown et al. 2016). The impact of MDD on the elderly population extends beyond mental health, as it can exacerbate co-existing medical conditions, increase the risk of disability, and contribute to higher mortality rates, particularly through suicide or complications arising from untreated conditions. Unfortunately, current pharmacological therapies for depression in older adults often result in suboptimal outcomes compared to younger populations, with notably low remission rates following initial treatment (Fu et al. 2019). This highlights a critical gap in effective management strategies for this vulnerable group. The treatment of depression in the elderly shows unique challenges due to the distinct epidemiological, phenotypic, and pathogenetic characteristics of the disorder in this population (Zhao et al. 2023). Moreover, the use of certain antidepressant classes in older adults has been associated with a higher incidence of adverse effects, particularly gastrointestinal and respiratory complications, when compared to placebo. These factors underscore the urgent need for tailored therapeutic approaches and innovative interventions to improve outcomes in geriatric depression (Banerjee et al. 2011). Furthermore, no single mechanism fully explains the aetiology of depression, which complicates the development of effective pharmacological therapies. Depression is a multifactorial condition involving neurobiological, genetic, environmental, and psychological components (Alshaya 2022). Dysregulation of neurotransmitter systems (e.g., serotonin, dopamine, and norepinephrine pathways), alterations in neuroplasticity, hypothalamic-pituitary-adrenal axis dysfunction and inflammatory processes have all been implicated. However, their relative contribution varies among individuals, highlighting the need for personalised treatment approaches and continued research into the complex pathophysiology of depression to refine therapeutic strategies.

Nowadays, there is a growing interest in the investigation of substances broadly categorised as ‘psychedelics’, with particular attention to their potential therapeutic applications in psychiatric disorders. Prominent examples of this class of compounds include psilocybin, a naturally occurring substance derived from fungi of the *Psilocybe* genus, and lysergic acid diethylamide (LSD), a synthetic compound originally derived from ergot alkaloids. Other well-known psychedelics include mescaline, a naturally occurring compound found in cacti such as peyote and San Pedro, and DMT (dimethyltryptamine), a tryptamine-based psychedelic found in various plants and animals. Additionally, compounds like ayahuasca, a traditional Amazonian brew containing DMT, and 2 C-B, a synthetic phenethylamine, are increasingly studied for their neuropsychological effects and therapeutic potential. Emerging evidence from clinical trials indicates that these compounds may exert significant effects in mitigating affective symptoms across a diverse range of neuropsychiatric disorders (Andersen et al. 2021).

This paper is focused on highlighting the potential of classic psychedelics as a novel therapeutic approach for major depressive disorder (MDD), particularly in elderly individuals burdened by age-related comorbidities. Extensive research has identified several underlying mechanisms contributing to depression, including neuroinflammation, dysregulation of the glutamatergic and serotonergic systems (Mann 1999; Mathews et al. 2012; Alexopoulos 2019). Psychedelics, with their proven anti-inflammatory properties and capacity to modulate these neural pathways, offer a multifaceted strategy for addressing depression. Investigating these substances in the context of ageing may bridge existing theoretical models with practical clinical applications, targeting both the psychological and systemic effects of ageing (Husain et al. 2022; Nichols, 2022).

Exploring the therapeutic potential of psychedelics in older adults presents a valuable opportunity to enhance our understanding of age-related depression while expanding the available therapeutic options in geriatric care. We strongly advocate for continued research in this promising field, as it represents a scientifically grounded, innovative approach that could significantly improve both the quality of care and the mental well-being of elderly individuals suffering from depression.

## Exploring classical psychedelics as potential antidepressants

Classical psychedelics (CP) have a long history of spiritual and therapeutic use, laying the groundwork for their resurgence in modern medical research. Scientific interest in CP expanded after the isolation of mescaline in 1897, and further grew following Albert Hofmann’s synthesis of LSD in 1938 and subsequent discovery of its psychoactive effects in 1943. This led to decades of research into their therapeutic potential (Doblin et al. 2019). Clinical studies from the 1950s and 1960s, along with modern research, have highlighted CP’s promising applications in treating psychiatric disorders, such as alcohol addiction and end-of-life distress in cancer patients, through psychedelic-assisted psychotherapy.

Psilocybin (PS), a naturally occurring compound from the tryptamine class of hallucinogens, has shown promising therapeutic potential in clinical studies targeting psychiatric disorders, particularly within the depressive spectrum, such as anxiety and depression in cancer, treatment-resistant depression, and substance abuse (Johnson and Griffiths 2017). Notably, PS administration has been found to significantly alleviate symptoms of treatment-resistant depression and major depressive disorder, positioning it as a potential innovative antidepressant treatment. Its mechanism of action, mainly through serotonin 5-HT2A receptor agonism, is believed to promote neural plasticity and facilitate profound psychological insights, contributing to its therapeutic effects (Carhart-Harris et al. 2016; Davis et al. 2021). Although the exact mechanisms in psychiatric conditions remain not fully understood, PS has been shown to enhance mood, increase empathy, and reduce negative affective states at doses of approximately 15 mg/kg or 20–30 mg for a 70 kg individual. Research on psilocybin and LSD highlights two dosing strategies: a single high-efficacy dose and microdosing, which involves sub-hallucinogenic doses taken chronically, around 10% of a full hallucinogenic dose, several times a week (Kuypers 2020; Davis et al. 2021). Despite its growing popularity, particularly among recreational users, the therapeutic benefits of microdosing in depressive disorders remain underexplored. A recent study of 278 participants engaged in psilocybin microdosing reported improvements in mood, increased happiness, and optimism, as well as reductions in depressive symptoms. However, these benefits were more frequent than other reported effects, emphasising the need for further controlled clinical research to validate microdosing as a viable therapeutic strategy (Anderson et al. 2019). Clinical studies involving healthy participants have demonstrated a favourable safety profile for PS and LSD when administered under controlled conditions. These substances have not been widely associated with long-term toxic effects, particularly in the cognitive and neurological domains, and exhibit a low potential for addiction. Safe administration is ensured by strict dosing protocols, closely monitored frequency of use, and psychological support to manage acute effects if necessary (Kuypers 2020; Davis et al. 2021). A recent prospective cohort study examining the effects of guided psychedelic group sessions on the well-being of older adults found clinically significant improvements primarily in individuals with underlying psychiatric conditions. Notably, the study reported no substantial differences in the positive effects of psychedelic therapy between older and younger adults (Kettner et al. 2024).

LSD, another prominent psychedelic from the tryptamine class, has shown high potential for modulating mood disorder symptoms. At doses ranging from 75 to 150 µg, LSD induces acute alterations in consciousness, including euphoria, dreamlike effects, enhanced introspection, and sensory changes. While higher doses (100 µg) may impair attention and concentration, these cognitive disruptions do not persist beyond the acute phase, and learning processes remain unaffected at doses up to 150 µg. Importantly, LSD has demonstrated a favourable safety profile, with no evidence of harm to internal organs, and minimal neuropsychological disturbances when used in controlled settings (Passie et al. 2008). Despite these promising findings, legal restrictions on LSD limit its therapeutic exploration (Fuentes et al. 2019). Pharmacodynamically, LSD’s interaction with central serotonin receptors positions it as a valuable candidate for research into psychiatric disorders. However, most studies to date have focused on patients with life-threatening conditions, leaving a gap in data regarding its efficacy in MDD (Holze et al. 2023). Future studies are essential to clarify LSD’s role in treating MDD and expand its potential applications. In a randomised, double-blind, placebo-controlled study, LSD administered in two sessions (200 µg each) resulted in lasting reductions in anxiety and depressive symptoms for up to 16 weeks. These results suggest that CP could be an effective treatment option for elderly patients, particularly if administered with extended breaks between doses. This approach could improve treatment adherence and reduce adverse effects from polypharmacy, offering significant benefits for depression treatment in older populations. These findings highlight the potential of classical psychedelics to address unmet needs in psychiatric care and pave the way for their inclusion in future therapeutic strategies (Holze et al. 2023).

## Molecular insights into the therapeutic effects of psychedelics in depression

Understanding the mechanism of action of psychedelics in MDD is grounded in the current knowledge of the pathophysiology of this persistent mood disorder. Evidence to date underscores the pivotal role of serotonin (5-hydroxytryptamine, 5-HT) in modulating the symptoms associated with MDD. The serotonergic system is well established as a key regulator of mood, sleep, and attention. Notably, most studies report a reduction in the density of 5-HT1A serotonin receptors in the prefrontal cortex, anterior cingulate cortex and temporal cortex, which is clinically associated with heightened anxiety levels. Conversely, post-mortem analyses of individuals with suicidal tendencies in the context of MDD have revealed an inverse relationship between elevated 5-HT2A receptor density in the prefrontal cortex and decreased extracellular serotonin levels. This molecular mechanism appears to be closely linked to the manifestation of pessimistic attitudes characteristic of depressive states. Collectively, these findings highlight the critical role of serotonin receptors in the pathophysiology of MDD and underscore their potential as therapeutic targets for mood disorders (Kometer et al. 2012; Meyer 2012).

**Figure 1.**
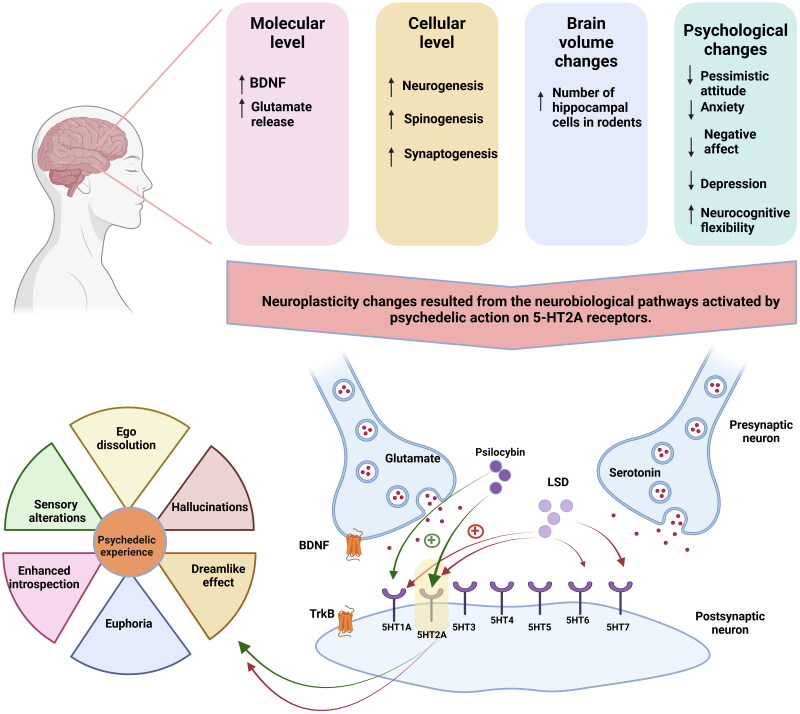
Activation of 5HT receptors by psilocybin and LSD: Implications for neuroplasticity and therapeutic potential in major depressive disorders. The activation of 5-HT2A receptors by psilocybin and LSD induces a cascade of neuropsychological changes in both humans and rodent models. A key advantage of this receptor-mediated mechanism is the enhancement of brain neuroplasticity. The neuroplasticity model of depression posits that disruptions in the brain’s self-regulatory mechanisms occur at various levels, including cellular, molecular and psychological domains. Psychedelics appear to counteract these disruptions, thereby restoring neuroplasticity. LSD administration has been shown to elevate serum levels of BDNF, a finding corroborated by a clinical study conducted on healthy human subjects, where significant increases were observed at 4- and 6-hour post-administration. BDNF binds to TrkB receptors, activating intracellular signalling pathways critical for neuronal survival and differentiation. Additionally, the activation of 5-HT2A receptors expressed on glutamatergic pyramidal neurons, particularly in cortical layers V and VI, mediates the effects of both LSD and psilocybin on serotoninergic and glutamatergic neurotransmission. Psilocybin has demonstrated potential in promoting synaptogenesis in patients with mild cognitive impairment. Furthermore, both LSD and psilocybin enhance neurogenesis and synaptogenesis, positioning these compounds as promising therapeutic candidates for neurodegenerative conditions, particularly in elderly populations. Finally, the psychological effects induced by psychedelics have been extensively observed in both clinical settings and preclinical models of depression. These findings highlight their multifaceted therapeutic potential, encompassing neuroplasticity enhancement and psychological symptom relief (Artin et al. 2021; de Vos et al. 2021; Kozlowska et al. 2022; Song et al. 2023). Abbreviations: brain-derived neurotrophic factor- BDNF; tropomyosin receptor kinase B- TrkB; lysergic acid diethylamide-LSD; 5-hydroxytryptamine receptors-5HT.

The mechanism of action of classical psychedelics remains a subject of ongoing investigation. Revolutionary advancements in imaging techniques and highly standardised diagnostic tools for assessing mental states have provided valuable insights into the brain’s neural circuits and their influence on mental states following the administration of these psychoactive substances. From a pharmacodynamic perspective, psychedelics primarily exert their effects through modulation of the serotonin system. A key feature of many psychedelics is their agonistic action on the 5-hydroxytryptamine 2 A (5-HT2A) receptors, which plays a central role in the observed effects. The acute subjective effects of psychedelics, such as hallucinations, visual distortions, dreamlike experiences, and mood enhancement, are largely attributed to their agonistic activity on serotonin receptors (Kometer et al. 2012). While the precise contribution of individual serotonin subreceptors to these effects remains incompletely understood, recent research has shown that psilocybin, through its active metabolite psilocin, activates not only 5-HT2A but also 5-HT1A and 5-HT2C subreceptors. A similar serotonin receptor agonism is observed in other hallucinogens, including DMT, mescaline, and related compounds. This complex multi-receptor activation mechanism underlies both the therapeutic effects (antidepressant, anxiolytic) and potentially negative effects, making the study of these compounds challenging (Figure 1). Additionally, psilocybin appears to improve mood not only through serotonin receptor modulation but also by enhancing neurocognitive flexibility and promoting neuroplasticity in prefrontal-limbic circuits (Kometer et al. 2012; Doss et al. 2021).

Beyond their shared mechanism of action, psychedelics may also interact with other receptor systems, adding complexity to their effects and contributing to the diversity of experiences they elicit. For example, LSD has a high affinity for several serotonergic receptor subtypes, including 5-HT1A, 5-HT1B, 5-HT1D, 5-HT1E, 5-HT6, and 5-HT7, as well as dopamine receptors (D1, D2, D3, D4, and D5). It acts as a partial agonist at 5-HT2A receptors, primarily found on neocortical pyramidal cells (Andersen et al. 2021). LSD also engages α1 and α2 adrenergic receptors and activates trace amine-associated receptor 1 (TAAR1), a promising target for addiction treatment. This broad receptor activity likely accounts for the diverse acute effects reported in humans (Passie et al. 2008; Simmler et al. 2016; McClure-Begley and Roth, 2022).

In the context of mood disorders, such as MDD, LSD’s therapeutic effects are largely attributed to its agonistic action at 5-HT2A receptors. Animal studies have shown that both LSD and psilocybin induce downregulation of 5-HT2A receptors (Buckholtz et al. 1988), although caution is warranted when extrapolating these findings to humans due to species-specific differences in receptor structure (McClure-Begley and Roth, 2022). Furthermore, the lack of selectivity of classical psychedelics for 5-HT2A receptors, which would be ideal for treating psychiatric disorders, leads to off-target effects, especially on serotonin receptor subtypes like 5-HT2B. Chronic activation of 5-HT2B has been linked to an increased risk of valvular heart disease, as observed with prolonged use of MDMA and certain Parkinson’s medications. While this risk has not been specifically studied with classical psychedelics, it is an important consideration for elderly patients with MDD (Droogmans et al. 2007; Zanettini et al. 2007).

The agonistic effects of psychedelics on 5-HT2C receptors, which can induce anorexia, also pose particular risks for older adults. Anorexia is a common condition in ageing populations and a major predictor of morbidity and mortality (Landi et al. 2016; McClure-Begley and Roth, 2022). This risk should be carefully weighed in the use of psychedelics for older individuals. Moreover, a thorough discussion is needed on the potential for serotonin syndrome, particularly in older adults, where polypharmacy may exacerbate this life-threatening condition (Poeschla et al. 2011).

In addition to these serotonergic effects, age-related physiological changes can significantly influence the safety and efficacy of psychedelics in the elderly. As individuals age, several physiological processes undergo changes that impact how the body processes drugs. For example, hepatic metabolism and renal excretion, both crucial for the elimination of many substances, tend to decline with age (Mangoni and Jackson 2004; Ngcobo 2025). These changes, along with alterations in body composition and plasma protein binding, can modify the pharmacokinetics of psychedelic compounds. As a result, the drug concentrations and effects in elderly individuals may be more pronounced and prolonged, potentially increasing the risks of adverse effects, including serotonin syndrome and other drug interactions (Drenth-van Maanen et al. 2019; Zazzara et al. 2021). Absorption of psychedelic compounds can also be influenced by age-related changes in the gastrointestinal system. While ageing does not substantially alter drug absorption, factors such as comorbidities, drug-specific properties, and diet can modulate this process, especially in psychedelic compounds. For drugs utilising active transport mechanisms, reduced absorption in older adults may result from decreased gastric motility. Altered gastric pH can also affect weakly basic drugs, particularly in conditions like atrophic gastritis, where hypochlorhydria impairs absorption (Drenth-van Maanen et al. 2019; Ngcobo 2025). Psilocybin, requires acidic conditions for conversion to psilocin, its active form. Reduced first-pass metabolism may further alter prodrug absorption, resulting in higher plasma concentrations of inactive forms without central effects (MacCallum et al. 2022). Psilocybin’s oral bioavailability is around 50%, with peak plasma concentrations of psilocin occurring 2–3 h post-ingestion in humans. In rodents, its half-life is approximately 0.9 h, indicating caution when extrapolating animal data to humans. In contrast, DMT has low oral bioavailability due to extensive MAO-A degradation, requiring an MAO-A inhibitor for therapeutic effects (Kelmendi et al. 2022).

Age-related changes in body composition, such as increased fat mass and decreased body water, influence the pharmacokinetics of lipophilic psychedelics like LSD and psilocybin, resulting in a higher volume of distribution, prolonged half-life, and prolonged effects. Reduced plasma protein levels (e.g., albumin) may enhance the pharmacodynamic effects of highly protein-bound psychedelics (Drenth-van Maanen et al. 2019). Hepatic metabolism changes with age, particularly phase I reactions (oxidation, reduction, hydrolysis) mediated by CYP450 enzymes. Phase II metabolism (glucuronidation, sulphation, acetylation) remains largely unaffected, suggesting that psychedelics primarily eliminated *via* phase II pathways are less impacted by ageing (Drenth-van Maanen et al. 2019). Psilocybin is rapidly dephosphorylated to psilocin in the stomach or by enzymes like alkaline phosphatase in various tissues. Psilocin is metabolised *via* glucuronidation and oxidation in the liver, though the exact role of CYP450 enzymes is unclear. However, CYP enzymes are known to metabolise other psychedelics, including LSD, DMT, and MDMA. Genetic polymorphisms in CYP3A4, CYP2C9, and CYP2C19 can affect plasma concentrations of LSD, which is converted to active metabolites O-H-LSD and nor-LSD, potentially altering its clinical effects (Gallo et al. 2019; Luethi et al. 2019).

Excretion, primarily *via* the kidneys, is affected by age-related changes such as reduced kidney size, increased fibrosis, tubular atrophy, and decreased glomerular filtration rate (GFR) (Drenth-van Maanen et al. 2019). Comorbid conditions, especially cardiovascular and metabolic diseases, worsen these effects, necessitating dosage adjustments. Serum creatinine-based GFR estimation remains the preferred method for evaluating renal function, despite limitations due to decreased muscle mass in elderly individuals. Psychedelics are largely eliminated through renal excretion. For psilocybin, a minor portion (∼1.5%) is excreted as unconjugated psilocin. Urinary metabolites include psilocin-O-glucuronide (20%) and 4-HIAA (33%), along with other less-characterised metabolites (Thomann et al. 2024). The potential for impaired renal clearance underscores the importance of careful dosing in elderly patients. The high lipophilicity of psychedelics facilitates blood-brain barrier penetration, contributing to their central effects. Their structural similarity to serotonin underlies their affinity for serotonin receptors, a key factor in their neurobiological activity.

## Preclinical models and experimental approaches

To date, a wide range of behaviours induced by psychedelics has been observed in rodent models, particularly in mice and rats. Among these, two behavioural paradigms are most commonly employed to characterise the pharmacological action of psychedelic substances: drug discrimination (DD) and the head twitch response (HTR) (Halberstadt et al. 2020). DD or drug substitution is a highly pharmacologically specific assay. It is widely used to evaluate the ability of test compounds to elicit hallucinogen-like effects in animals. Moreover, DD enables researchers to analyse and compare the relative potencies of various hallucinogenic substances, providing critical insights into their pharmacodynamic profiles (Halberstadt and Geyer 2013).

HTR is a behavioural assay for 5-HT2 receptors agonists and is clinically manifested by rapid and rhythmic side-to-side rotational head movements in mice and rats (Halberstadt and Geyer 2018). The role of 5-HT2 receptor activation in eliciting the HTR has been well-documented in preclinical studies, despite evidence suggesting that multiple neurological pathways contribute to this behaviour. HTR is widely recognised as a marker that distinguishes hallucinogenic from non-hallucinogenic agonists. However, exceptions to this pattern exist, as certain non-hallucinogenic compounds have been shown to induce HTR in rodents without producing hallucinogenic effects in humans. Examples include the non-selective 5-HT agonists quipazine and lisuride. Quipazine, an arylpiperazine, has been studied for its potential antidepressant effects, while lisuride, a structural analogue of LSD, is utilised in the treatment of Parkinson’s disease (Zanettini et al. 2007). These inconsistencies underscore limitations in the predictive validity of HTR for evaluating the hallucinogenic properties of compounds. Nonetheless, it remains clear that all hallucinogenic substances consistently elicit a positive HTR. For instance, selective 5-HT2A antagonists effectively block the HTR induced by hallucinogens such as LSD, psilocybin, MDMA and others (Wang et al. 2017). For example, selective 5-HT2A antagonists have been shown to block the head twitch response (HTR) induced by LSD, psilocybin, MDMA and other hallucinogens (Hesselgrave et al. 2021). Animal models have further been employed to investigate the activation of specific serotonergic subreceptors. In this context, compelling evidence supports the involvement of cortical 5-HT2A receptors, labelled with [3H]-Ketanserin, in the expression of HTR behaviour (Halberstadt et al. 2020). Additionally, hallucinogenic substances fail to induce HTR in 5-HT2A knockout mice, underscoring the critical role of this receptor subtype. However, the HTR test has notable limitations, including its inability to assess responses over extended periods and the reliance on human subjectivity in observational assessments, which increases the likelihood of error (Naismith et al. 2012; Halberstadt and Geyer 2013). These 2 behavioural tests provide important information about the hallucinogenic effects of psychedelics, but do not help assess antidepressant response. However, there are several challenges when we use preclinical animal models in psychiatric pathology. For the study of antidepressant effects, the tendency is to use standardised behavioural models to study effects particular to the depressive phenotype, such as: anhedonia (the sucrose preference test), behavioural despair (the forced swim test, tail suspension test), hopelessness (shock avoidance test with measure of escape latency), anxiety-like behaviour (elevated plus maze) or analysis tests of weight gain/loss (Wang et al. 2017). Behavioural tests are extensively utilised to evaluate the antidepressant effects of these compounds administration in animal models, both independently and in conjunction with hallucinogenic response assays. A notable example comes from a recent study demonstrating that a single dose of psilocybin reversed anhedonic behaviour in the sucrose preference test in mice (Hesselgrave et al. 2021). Interestingly, this effect was observed even in the absence of HTR when 5-HT2 receptors were blocked. These findings suggest the potential for psilocybin to be used as an antidepressant in drug combinations designed to suppress its hallucinogenic effects. Consequently, preclinical models offer valuable opportunities to enhance current understanding, particularly when used in combination to explore these mechanisms. Moreover, these findings might be applicable to the treatment of depression associated with ageing, as they offer the potential to avoid serotonin syndrome, a condition frequently encountered in the elderly population undergoing polypharmacy for mood disorders. Additionally, there are several limitations in current animal models of depression that hinder their ability to fully replicate the complexity of human depression. One of the primary limitations is the oversimplification of the disorder. Human depression is multifactorial, influenced by genetic, environmental, psychological, and social factors, which are difficult to model accurately in animals. While animal models can replicate certain symptoms, such as anhedonia or behavioural despair, they do not capture the broader spectrum of human depression, including the intricate interplay of environmental stressors, trauma, or social contexts that contribute to the condition.

## Clinical investigations of classical psychedelics in elderly populations

In general, psychedelics such as psilocybin and LSD have demonstrated the ability to induce rapid and sustained improvements in depression, with effects sometimes lasting for several months. These outcomes have often been more pronounced than those associated with traditional antidepressants, which typically require several weeks to produce noticeable improvements. However, the age-specific effects and safety of psychedelics in older adults, particularly those with comorbid conditions, remain insufficiently studied. The most relevant findings regarding the effects of psychedelics in the adult population, including age-related considerations, are summarised in Table 1.

**Table 1.** Therapeutic role of psychedelics in adults with depression: a focus on ageing populations.

Therapy	Study design and participants	Findings	References
Ayahuasca or placebo (a single dose of 1 mL/kg)	double-blind, parallel-arm, randomised, placebo-controlled trial (*N* = 27); adults aged 18–60 years with treatment-resistant depression;	The primary outcome was the change in depression severity, assessed using the HAM-D scale from baseline to 7 days (D7) after dosing, while the secondary outcome focused on the change in MADRS scores at 1 (D1), 2 (D2), and 7 (D7) days post-dosing.	(Palhano-Fontes et al. 2019)
Two experimental psilocybin sessions (session 1: 20 mg/70 kg; session 2: 30 mg/70 kg)	randomised clinical trial (*N* = 24); participants aged between 21 to 75 years with moderate or severe MDD episodes, as assessed using the SCID-5 and the GRID-HAMD scales; no current pharmacotherapy for depression at the time of trial screening; without histories of psychotic disorder or serious suicide attempt.	Clinically, 71% of participants achieved *a* ≥ 50% reduction in GRID-HAMD scores by weeks 1 and 4, and remission rates were 58% at week 1 and 54% at week 4, highlighting the intervention’s robust and sustained antidepressant effects.	(Davis et al. 2021)
Psilocybin versus escitalopram two doses of 25 mg psilocybin, 3 weeks apart, plus 6 weeks of daily placebo; two doses of 1 mg psilocybin, 3 weeks apart, plus 6 weeks of daily oral escitalopram	6-week follow-up phase 2, double-blind, randomised, controlled trial (*N* = 59); patients (18–80 years old) with long-standing, moderate-to-severe MDD;	Primary outcome: Change in the score on the 16-item Quick Inventory of Depressive Symptomatology–Self-Report (QIDS-SR-16; range 0–27, higher scores indicate greater depression) at week 6. Secondary outcome: QIDS-SR-16 response (reduction in score >50%) at week 6.	(Carhart-Harris et al. 2021)
Psilocybin versus escitalopram 25 mg of psilocybin + psychological support 10 mg of escitalopram + psychological support	randomised clinical trial (*N* = 59); patients aged 18 to 80 years with, moderate to severe depression;	Psilocybin had a significantly greater impact on both thought suppression and rumination than escitalopram at the 6-week endpoint. Psilocybin responders showed reductions in both domains, while escitalopram responders only improved rumination, with no change in thought suppression despite meeting clinical response criteria.	(Barba et al. 2022)
A single dose of psilocybin (0.215 mg/kg body weight) or placebo, both administered with psychological support	double-blind, randomised clinical trial (*N* = 52); individuals aged 20–60 years diagnosed with MDD;	A single, moderate dose of psilocybin significantly reduced depressive symptoms compared to placebo, with effects lasting at least 2 weeks and no serious adverse events reported.	(von Rotz et al. 2023)
A single dose of psilocybin (25 mg) or niacin placebo and psychological support	placebo-controlled, 6-week randomised trial (*N* = 104); adults aged 21 to 65 years with MDD with symptoms present for at least 60 days;	Psilocybin with psychological support resulted in rapid, sustained reductions in depressive symptoms, without serious adverse events, suggesting its potential as a safe and effective antidepressant.	(Raison et al. 2023)
Psychedelic ceremony or retreats (involving use of psilocybin, LSD, ayahuasca, DMT/5-MeO-DMT, mescaline, or iboga/ibogaine)	psychiatric diagnoses included MDD, anxiety, alcohol dependence and ADHD; 62 older adults (age ≥65 years) and 62 matched younger adults	Older adults showed similar well-being improvements to younger participants after a psychedelic retreat, even though they reported less intense immediate effects. Unlike younger individuals, the positive changes in older adults were more strongly associated with feelings of connection and shared experiences during group activities, rather than the immediate intensity of the psychedelic experience itself.	(Kettner et al. 2024)
Psilocybin versus escitalopram two oral doses of 25 mg psilocybin with psychological support; daily oral escitalopram: 10 mg for 3 weeks, increased to 20 mg for the subsequent 3 weeks, psychological support.	6-month follow-up of a phase 2, double-blind, randomised, controlled trial (*N* = 59); patients with moderate-to-severe MDD; aged 18–80 years, with no MRI or SSRI contraindications;	After 6 months of follow-up, both psychological and experimental therapy led to comparable reductions in depressive symptoms (QIDS-SR-16). However, psilocybin showed significantly greater long-term benefits in work and social functioning, connectedness and meaning in life. These findings suggest that while both therapies are effective in reducing depressive symptoms, psilocybin may offer superior and more sustained improvements in overall mental health.	(Erritzoe et al. 2024)

MDD, Major Depressive Disorder; SCID-5, Structured Clinical Interview for Diagnostic and Statistical Manual of Mental Disorders-5; GRID-HAMD, GRID-Hamilton Depression Rating Scale; LSD, lysergic acid diethylamide; DMT/5-MeO-DMT, dimethyltryptamine/5-methoxy-dimethyltryptamine; QIDS-SR-16, 16-item Quick Inventory of Depressive Symptomatology Self-Report; HAM-D, Hamilton Depression Rating Scale; MADRS, Montgomery-Åsberg Depression Rating Scale.

In older adults, pharmacokinetic and pharmacodynamic properties differ significantly from those of younger individuals, particularly due to the high prevalence of polypharmacy. For many medications, including antidepressants, lower initial doses and gradual titration are recommended. However, it remains unclear whether psychedelics, such as psilocybin, should follow similar protocols. A post hoc analysis of pooled data from psilocybin studies found no significant relationship between age or weight and subjective effects, although the impact on adverse events was not assessed. Additionally, the oldest participant in this analysis was 71, and the overall sample consisted of relatively healthy individuals, which limits generalisability to older populations (Garcia-Romeu et al. 2021).

Polymedication, a common practice in geriatric care, raises concerns regarding drug interactions. Therefore, a comprehensive evaluation of the risks associated with combining psychedelic substances with other medications is essential. Pharmacokinetic interactions, particularly those involving enzymes responsible for metabolising psychedelics, should be carefully considered. For example, the enzyme monoamine oxidase (MAO) plays a crucial role in the demethylation and oxidative deamination of psilocybin. When MAO inhibitors are co-administered, these processes may be disrupted. Some individuals deliberately combine MAO inhibitors with psilocybin to prolong its psychoactive effects by delaying its elimination (Halpern, 2004). Additionally, co-administration with ethanol can extend psilocybin’s hallucinogenic effects, potentially through the formation of tetrahydroisoquinolines and β-carbolines, metabolites that inhibit MAO activity. Tobacco uses also reduces central and peripheral MAO activity, further enhancing the effects of psychedelics (Dinis-Oliveira 2017). In contrast, combining MDMA with MAO inhibitors like phenelzine—commonly prescribed to elderly patients with depression—can delay metabolite clearance by modulating cytochrome P450 enzymes (CYP1A2, CYP2C19, CYP2D6, and CYP3A4). This can lead to adverse events such as hypertension, diaphoresis, and altered mental status. Similarly, combining psilocybin with haloperidol reduces MAO activity, potentiating the psychomimetic effects of psilocin. In general, MAO and aldehyde dehydrogenase (ALDH) inhibitors tend to increase the toxicity of psilocybin while attenuating the effects of LSD. Furthermore, combining ayahuasca with fluoxetine, a selective serotonin reuptake inhibitor (SSRI), results in pharmacokinetic interactions that reduce the activity of CYP3A4, CYP2D6, and CYP2C19 enzymes, which can lead to adverse reactions such as tremors, confusion, and diaphoresis (Abbott et al. 2020; Halman et al. 2024; Ngcobo 2025).

Pharmacodynamic interactions are particularly important in elderly patients, as psychedelics primarily act on the central serotonin system. While there is a theoretical risk of serotonin syndrome when multiple agents simultaneously target serotonin receptors, a recent systematic review supports the safe co-administration of psychedelics like psilocybin with other serotonergic drugs (Halman et al. 2024). Additionally, common medications used by older adults, such as beta-blockers for cardiovascular conditions, should be considered in this context. For example, combining DMT with pindolol, a beta-blocker, appears to enhance the effects of DMT, likely due to pindolol’s affinity for both 5HT1A and β-adrenergic receptors. Similarly, reserpine, an antihypertensive agent, may prolong the adverse effects of LSD, such as tremors, through pharmacodynamic interactions (Halman et al. 2024). Further concerns arise regarding the cardiovascular safety of psychedelics in older adults, who are more prone to hypertension, coronary artery disease, and cerebrovascular events. High-dose psilocybin has been associated with an increased risk of moderate-to-severe hypertension in older patients with complex medical profiles. Additionally, psilocybin demonstrates a dose-dependent increase in the QTc interval; however, this effect is generally considered clinically insignificant at doses ≤30 mg (Dahmane et al. 2021). Nonetheless, pre-treatment cardiovascular screening and continuous monitoring during sessions remain essential to mitigate potential adverse events. Moreover, metabolic disorders prevalent among elderly individuals, such as diabetes mellitus and obesity, can significantly influence the pharmacokinetics and pharmacodynamics of psychedelic compounds. Alterations in drug metabolism and distribution resulting from impaired hepatic or renal function—conditions more common in advanced age—necessitate cautious dose titration and close monitoring to avoid toxicity and enhance therapeutic efficacy (Halman et al. 2024; Ngcobo 2025). Cognitive impairments, including mild cognitive impairment (MCI) and early-stage dementia, present unique challenges when considering psychedelic therapy. While preliminary evidence suggests that psychedelics may enhance neuroplasticity and support cognitive resilience, these potential benefits must be carefully weighed against the risk of exacerbating confusion, anxiety, or perceptual disturbances in cognitively vulnerable patients. Ensuring appropriate patient selection and monitoring is critical to maximising therapeutic outcomes (Abdoli et al. 2022). These findings suggest that, although monitoring of comorbid conditions remains essential, the risks associated with psychedelics in elderly patients can be mitigated by adopting a tailored, patient-specific approach to treatment and rigorous monitoring. To achieve this, comprehensive patient screening is necessary, including detailed medical evaluations focused on cardiovascular health, liver and kidney function, and neurological status. Psychiatric assessments are crucial for identifying conditions such as schizophrenia or bipolar disorder, which may be exacerbated by psychedelics, while cognitive impairments must also be considered to ensure informed consent. Moreover, a thorough medication review is essential, particularly due to the high prevalence of polypharmacy in elderly patients, to identify any potential drug interactions, especially those that might involve serotonergic, dopaminergic, or cardiovascular systems (Ngcobo 2025). Age-related physiological changes also require careful attention when determining appropriate dosages. Elderly patients may experience altered pharmacokinetics and pharmacodynamics, making it necessary to start with lower doses than those typically used for younger populations. Dosing should be gradually adjusted based on individual tolerance, therapeutic response, and ongoing safety monitoring. Factors such as body mass, metabolic health, and comorbidities should guide these adjustments, ensuring that treatment remains both safe and effective. In addition to careful dosing, continuous monitoring throughout the treatment process is critical. During the acute phase, patients should be observed closely, especially if they have cardiovascular or psychiatric vulnerabilities. Monitoring should include vital signs, psychological status, and any signs of adverse reactions. Follow-up assessments are necessary post-treatment to track long-term safety, support psychological integration, and assess therapeutic outcomes. Emergency protocols should also be in place to address any acute adverse events or complications that may arise during or after treatment. Given the psychological intensity of psychedelic experiences, certified therapists should also be present to provide psychological support, especially for elderly patients who may be more vulnerable to anxiety or confusion during treatment (O’Donnell et al. 2019). Age-specific study designs, along with innovative tools such as virtual reality (VR), will be critical for capturing the full spectrum of risks and benefits in this demographic. In this regard, virtual reality (VR) technology offers a promising non-pharmacological alternative to psychedelics, particularly for elderly populations. VR has the potential to induce psychedelic-like experiences, such as altered states of consciousness and enhanced emotional processing, without the associated risks of drug interactions or cardiovascular risks. Emerging research has explored VR as a tool for creating immersive environments that stimulate cognitive functions, promote relaxation, and even induce hallucinatory-like perceptions similar to those produced by psychedelics (Aday et al. 2020; Magni et al. 2023). For older adults, VR presents several advantages as a customisable and non-invasive intervention. Unlike pharmacological psychedelics, which may be contraindicated due to the complex health profiles of elderly individuals, VR provides a safer and more controlled alternative. By adjusting the intensity, duration, and nature of the VR experience, clinicians can address the unique challenges posed by age-related cognitive decline, anxiety, or depression, while minimising physical risks. Additionally, VR has been shown to enhance neuroplasticity, creativity, and emotional regulation—key factors for elderly individuals facing conditions such as dementia, loneliness, or chronic illness. By complementing or supporting psychedelic therapies, VR may also provide a safer environment for administering these treatments.

## Conclusion and future perspectives

Research into the use of psychedelics for treating mood disorders in elderly populations holds great promise but presents unique challenges that require careful attention. The need to consider specific age subgroups and regional populations is paramount, as ageing trajectories, psychological profiles, and emotional well-being differ significantly across cultures. Studies have shown that subjective well-being varies by region, with more positive ageing outcomes observed in affluent Western societies compared to other areas such as Eastern Europe, sub-Saharan Africa, and Latin America (Alexander and Young 2023). These cultural differences highlight the importance of contextualising psychedelic research within diverse demographic and cultural frameworks to avoid biases that could skew results, especially regarding mood enhancement in older adults (Steptoe et al. 2015). Additionally, the heterogeneity within the elderly population, including those with varying levels of cognitive decline, physical health, and social support, must be considered when designing trials. Studies should not only focus on broad categories such as ‘elderly’ but also account for more granular subgroups, such as individuals with early-stage dementia versus those in later stages of ageing, as their responses to treatment may differ. Recent work has shown that the brain’s plasticity in older adults can be enhanced through psychedelics, which opens up new possibilities for cognitive rehabilitation and emotional regulation (Carhart-Harris et al. 2017). However, careful attention must be paid to the fact that elderly patients are often on multiple medications, which increases the risk of adverse interactions with psychedelics. As such, the safety profile of psychedelic compounds in older adults must be thoroughly assessed, especially with regard to cardiovascular, neurological, and psychiatric conditions common in ageing populations. Furthermore, given the complexity of using psychedelic compounds in elderly patients—due to the risk of adverse interactions with polypharmacy and the need for careful screening—administration of these substances should be preceded by thorough medical history assessments, including evaluating the pharmacodynamics and pharmacokinetics in older adults, which can differ significantly from younger populations. A careful approach that includes close monitoring during treatment, as well as the development of individualised dosing strategies, will be essential. Appropriate safety protocols must also include screening for contraindications such as a history of psychosis or other psychiatric conditions that could be exacerbated by psychedelic use. As the body of research continues to grow, this nuanced, patient-centred approach will be essential to unlocking the therapeutic potential of psychedelics for mood disorders in ageing populations while safeguarding against unforeseen risks. Future studies should not only aim to identify effective treatments but also prioritise minimising harm by adapting protocols to the unique needs and vulnerabilities of older adults. This includes establishing clear guidelines for psychedelic-assisted therapy, as well as promoting education and training for healthcare providers to safely incorporate these substances into clinical practice for ageing patients.
